# Genetic Damage Induced by a Food Coloring Dye (Sunset Yellow) on Meristematic Cells of *Brassica campestris* L.

**DOI:** 10.1155/2015/319727

**Published:** 2015-04-14

**Authors:** Kshama Dwivedi, Girjesh Kumar

**Affiliations:** Plant Genetics Laboratory, Department of Botany, University of Allahabad, Allahabad 211002, India

## Abstract

We have performed the present piece of work to evaluate the effect of synthetic food coloring azo dye (sunset yellow) on actively dividing root tip cells of *Brassica campestris* L. Three doses of azo dye were administered for the treatment of actively dividing root tip cells, namely, 1%, 3%, and 5%, for 6-hour duration along with control. Mitotic analysis clearly revealed the azo dye induced endpoint deviation like reduction in the frequency of normal divisions in a dose dependent manner. Mitotic divisions in the control sets were found to be perfectly normal while dose based reduction in MI was registered in the treated sets. Azo dye has induced several chromosomal aberrations (genotoxic effect) at various stages of cell cycle such as stickiness of chromosomes, micronuclei formation, precocious migration of chromosome, unorientation, forward movement of chromosome, laggards, and chromatin bridge. Among all, stickiness of chromosomes was present in the highest frequency followed by partial genome elimination as micronuclei. The present study suggests that extensive use of synthetic dye should be forbidden due to genotoxic and cytotoxic impacts on living cells. Thus, there is an urgent need to assess potential hazardous effects of these dyes on other test systems like human and nonhuman biota for better scrutiny.

## 1. Introduction

The food colouring history dates back to early Egyptians and Romans civilization, when people used saffron, various flowers, carrots, mulberries, beets, and so forth to put colour to their foods [1] suggesting use of coloring agents from prehistoric times. Later during the middle of the nineteenth century people had started using synthetic colors in place of natural colors [1]. Since then the extensive use of synthetic food azo dyes (–N=N–) has become very common due to increasing canned and fast food culture, despite their legislative ban.

Moreover these dyes have no nutritional value, they have no health benefits, they are not preservative [2]. They only make food attractive to meet new consumer demand, since the visual aspect is considered to be an important factor for the selection of products by final consumers [3].

As per norms of international research and the recommendations of the Codex Committee on Food Additives and Contaminants (CCFAC), intake of dye is under the control of ADI (acceptable daily intake) [4]. The maximum ADI established by Joint FAO/WHO Expert Committee on Food Additives (1994) for sunset yellow is 2.5 mg/kg [5]. Nowadays, food industries are ignoring the guidelines provided by these regulatory agencies to sell their products in a large scale.

Sunset yellow (molecular weight 452.36) is an azo dye, is orange yellow in color, and is permitted food color in India. It is extensively used in almost every type of food preparation like sweets, jams and jellies, soft drinks, candies, ice cream, canned juice, sauces, pickles, and so forth. In the past few years, use of some food dyes including sunset yellow was banned in United States and Japan owing to its mutagenicity which has been evidenced from several mammals bioassays [6]. However, in other countries like England, Brazil, and India, they were marketed freely due to their noncytotoxicity [7]. This suggests that the effect of dyes is not same for every individual; it may vary according to dose, age, gender, nutritional status, genetic factor [8], and most important on time of exposure.

Unlimited use of azo dye could be hazardous in sense of its adverse effects on human and nonhuman biota. Despite its important role in our food, azo dye could be serious threat to human health. Some azo dyes are metabolized in the intestinal wall and liver, producing free aromatic amines that are potentially carcinogenic and mutagenic [9–12]. Park et al. [13] stated that common colouring combinations of tartrazine, Red 40, Yellow 4, Yellow 5, Red 2, and brilliant blue FCF or Blue 1 had negative effects on the CNS (central nervous system) of human cell lines. Such combinations of food colourings are commonly used in processed foods.

Plant bioassays are quite sensitive and simple technique in comparison to animal bioassay to assess the genotoxicity and cytotoxicity of a chemical compound [14, 15]. Plant bioassays have been validated in international collaborative studies under the United Nations Environment Program (UNEP), World Health Organization (WHO), and US Environmental Protection Agency (US EPA) [16] and proved to be efficient tests for genotoxic monitoring of environmental pollutants [14, 17–19].

In literature ample studies are available on mutagenicity and clastogenicity of sunset yellow on several test systems [5, 6, 20, 21]. Yet adequate information on genetic damage potential of azo dye is still lacking. Generally in plant bioassay* Allium cepa* has been used as a model test system for genotoxic as well as cytotoxic effect of a chemical compound. Kumar and Srivastava [22] have used* Trigonella foenum graecum* to test the genotoxicity of sunset yellow on plant system. However, this is the first report using an important oilseed crop as a test material for monitoring cytotoxic and genotoxic efficiency of synthetic dye using mitotic index depression analysis.

## 2. Methodology

### 2.1. Materials and Methodology

#### 2.1.1. Procurement of Materials

Inbred seeds of cultivar* Brassica campestris* L. accession number IC363713 were obtained from National Bureau of Plant Genetic Resources (NBPGR) (IARI), New Delhi. Sunset yellow (C_16_H_10_N_2_Na_2_O_7_S_2_, molecular weight 452.36) used in present study has been purchased from Science Corporation, Allahabad, India. Manufacturer of sunset yellow is HiMedia Laboratory Private Limited, Mumbai, India (CAS No. 2783-94-0).

#### 2.1.2. Mitotic Preparation

For mitotic study homogenous and dry seeds were washed and then presoaked in distilled water for 5 hours. Presoaked seeds were placed in Petri plates layered with moistened Whatman filter paper and kept in incubator for germination at 25 ± 2°C. When roots of germinated seeds reached 2-3 cm in length they were treated with different concentrations of aqueous solution of sunset yellow, that is, 00% (control), 1%, 3%, and 5%, for 6-hours duration. After treatment roots were washed thoroughly to remove traces of dye and fixed in glacial acetic acid : alcohol (1 : 3 v/v). After 24 hours fixed material was transferred to 70% alcohol and stored at 4°C until use.

Fixed root tips were hydrolysed in 1 N-HCl for 5 min at 60 ± 2°C, washed with tap water to remove excess of HCl, and dried with blotting paper. Dried root tips were placed in watch glass containing 2% standard acetocarmine stain and kept for 45 min. After staining dark coloured apical tips were squashed in same stain by gentle thumb pressure. Slides were observed under* Nikon phase contrast research microscope (Nikon Eclipse E200, Japan)*. Approximately two-thousand-root tip cells were examined for each dose. Data for different cytological stages was indexed by mitotic index analysis (= number of dividing cells, divided by the total number of cells observed) whereas frequency of mitotic manifestations has been observed on the basis of total abnormality percentage (= number of abnormal cells, divided by the total number of cells observed).

#### 2.1.3. Statistical Analysis

Variations in the mean of MI and Ab. % were subjected to one-way analysis of variance (ANOVA) using post hoc multiple comparison from Tukey's test (*P* < 0.05) by using Statistica-8 software (Stat Soft). In all experiments, three replicates were performed for each dose. Data presented in terms of mean values and standard error (±SE).

### 2.2. Results and Discussion

*Brassica campestris* L. exhibits species level genomic constitution (2*n* = 20). Present assessment showed the normal course of mitotic division in the control set, that is, alignment of 20 chromosomes at metaphase and segregation of chromosomes into 20:20 at anaphase. In untreated meristematic cells (root tips), MI was registered to be 14.60% (±0.19) with no chromosomal manifestations. On the other hand, treated sets displayed the considerable range of irregularities during mitosis that were found to be distributed in almost all phases of division, that is, metaphase, anaphase, and telophase.

Table 1 presents the occurrence characteristics of normal and disturbed phases of cell cycle during mitotic cell cycles. Spectrum of mitotic manifestation is found to be dose dependent (Table 2). Conversely, increasing concentrations of dye (1%, 3%, and 5%) have induced the significant reduction (*P* < 0.05) in MI in a dose dependent manner (Table 3). Moreover some case of even cell death has been recorded at the 5% dose of azo dye. Lowering of MI might have been achieved by the inhibition of DNA synthesis at S-phase that most probably happened due to decreasing ATP level and the pressure from the functioning of the energy production centre [23, 24]. Hidalgo et al. [25] reported that the inhibition of certain cell cycle specific enzyme such as DNA polymerase, which is essential for DNA replication, might have caused antimitotic effect, resembling colchicine's mode of action. Colchicine inhibits the formation of spindle fibers and temporarily arrests mitosis [26]. This inhibition could be due to either the blocking of G_1_ suppressing DNA synthesis [27] or a blocking in G_2_ preventing the cell from entering mitosis [28, 29].

As a consequence of irregular mitosis, several aberrations were recorded, namely, precocious movement of chromosome, 3.11%, unorientation, 1.87%, C-mitosis, 1.41%, forward movement of chromosome, 1.67%, micronuclei formation at prophase and telophase, 5.42%, chromatin bridge, 0.22%, and stickiness of chromosomes, 7.58% at metaphase and 4.92% at anaphase. Among all the aberrations observed, stickiness was registered to be the highest followed by micronuclei formation. Moreover some other anomalies have also been recorded such as binucleate cell, unequal separation, and fragmentation.

Klasterska et al. [30] and McGill et al. [31] suggested that chromosome stickiness arises from improper folding of the chromosome fiber into single chromatids and the chromosomes become attached to each other by subchromatid bridges. Chromosome stickiness reflects highly toxic effects of mutagen, usually of an irreversible type probably leading to death [16]. This is in agreement with the present investigation, where cell death has been registered at the highest dose, thus clearly suggesting the cytotoxic impact of sunset yellow on test crop. Ahmed and Grant [32] mentioned that stickiness of chromosomes might have resulted from increased chromosome contraction and condensation.

In general, chromosomal aberrations are changes in chromosome structure resulting from a break or exchange of chromosomal material [33] and most often are permanent in nature. Further investigations showed the dominance of micronuclei after stickiness. Occurrence of micronuclei as aberration might be the results of acentric fragments or lagging chromosomes that fail to incorporate into either of the daughter nuclei during telophase of the mitotic cells [34, 35]. Thus, the micronuclei formation at telophase is attributed to genetic loss through genome elimination of chromosomes. Such genome loss plays a significant role in the production of aneuploids when occurring in germinal cells. Several hypotheses have been suggested in an attempt to explain the phenomenon, including inactivation of chromosomes by nuclease, formation of multipolar spindles, asynchrony in nucleoprotein synthesis, genome ratios, spatial separation of genomes, and suppression of centromere function in the eliminated chromosomes, asynchronous cell cycle phases, and asynchronous mitotic and meiotic rhythms [36]. However, more precise explanation is still lacking.

Phenomenon of C-mitosis was first reported by Levan [37] in root meristems of* Allium cepa* L. as disruption of the spindle fibres leading to the random scattering of the condensed chromosomes. Badr [38] suggested that C-mitosis is the indication that the food colorant has inhibited spindle formation similar to the effect of colchicine. Induction of C-mitosis commonly is associated with spindle poisons, indicating tubogenic effect [37]. Our study is in agreement with the findings of many researchers [16, 24, 39–47]. They also reported the mitoinhibitory potential of food additives and some related chemicals on higher plants such as* Vicia faba*,* Allium cepa* L., and* Trigonella foenum graecum* because higher plants provide a useful genetic system for screening and monitoring the potency of environmental hazards.

## 3. Conclusion

Present findings suggest the genotoxic and cytotoxic activity of food colouring dye on the cell cycle of* B. campestris* L. All three doses showed the considerable decrease in MI along with the increasing doses. However, highest dose of dye, that is, 5%, had shown severe cytotoxicity in terms of cell death. So, present finding clearly depicts the genotoxic and cytotoxic impact of sunset yellow on actively dividing root tip cells of* Brassica campestris* L. This investigation is also in agreement with several previous studies in the literature suggesting that there is an urgent need to assess potential hazardous effects of azo dyes on other test systems like human and nonhuman biota for better scrutiny.

## Figures and Tables

**Table 1:** Occurrence of normal and disturbed phases of cell cycle in meristematic root tip cells of *Brassica campestris* L. after treatment with sunset yellow.

	Treatments	Normal metaphase	Normal anaphase	Normal telophase	Disturbed metaphase	Disturbed anaphase	Disturbed telophase
Replicate 1	Control (distilled water)	**+**	**+**	**+**	**−**	**−**	**−**
Replicate 2		**+**	**+**	**+**	**−**	**−**	**−**
Replicate 3		**+**	**+**	**+**	**−**	**−**	**−**

Replicate 1	1% dye	**−**	**+**	**+**	**+**	**−**	**−**
Replicate 2		**+**	**+**	**+**	**+**	**−**	**−**
Replicate 3		**+**	**+**	**+**	**−**	**+**	**−**

Replicate 1	3% dye	**+**	**+**	**+**	**−**	**−**	**+**
Replicate 2		**+**	**+**	**−**	**−**	**+**	**−**
Replicate 3		**−**	**+**	**+**	**+**	**+**	**+**

Replicate 1	5% dye	**−**	**+**	**+**	**−**	**+**	**+**
Replicate 2		**−**	**−**	**−**	**+**	**+**	**+**
Replicate 3		**+**	**−**	**−**	**+**	**+**	**+**

**Table 2:** Frequency of mitotic abnormalities (at metaphase, anaphase, and telophase) in azo dye treated root tip meristems of *Brassica  campestris *L.

Treatments %	Metaphase abnormalities %					Anaphase abnormalities %					Telophase abnormality (%)^***^
	Pm	Un	C-mt	St	Oth^*^	Bg	Lg	Fm	St	Oth^**^	
Control	—	—	—	—	—	—	—	—	—	—	—
1	—	0.01	—	1.48	0.6	0.01	—	0.17	1.0	—	1.50
3	1.09	0.32	0.30	2.80	0.29	—	0.35	0.71	1.33	0.19	1.75
5	2.02	1.54	1.11	3.30	0.47	0.21	0.51	0.79	2.59	0.67	2.17

Pm: precocious movement of chromosomes, Un: unorientation, C-mt: C-mitosis, St: stickiness, Bg: bridge, Lg: laggards, Fm: forward movement of chromosomes, Oth: other abnormalities, ^*^clumping, ^**^fragmentation, unequal separation, and binuleate cells, and ^***^micronuclei.

**Table 3:** Reciprocal relationship between MI (%) and total abnormalities (%) along with the increasing doses.

Dye treatments	Total cells observed	^**^T Ab. (%)	Mitotic index (MI) (%)
	(mean ± SE)	(mean ± SE)	(mean ± SE)
Control	1715 ± 9.64	00.00^a∗^	14.60 ± 0.19^a∗^
1%	1700 ± 5.85	3.57 ± 1.10^a^	12.32 ± 0.40^a^
3%	1748 ± 8.37	7.28 ± 0.20^b^	7.88 ± 0.12^b^
5%	1663 ± 12.01	12.80 ± 0.39^c^	3.47 ± 0.51^c^

^*^Mean values designated by different lowercase letters differ significantly at the 0.05 level by Tukey test; ^**^T Ab. indicate total abnormalities.
